# Supplementary material for: APOBEC3-Mediated RNA Editing in Breast Cancer is Associated with Heightened Immune Activity and Improved Survival
Source: Int J Mol Sci. 2019 Nov 10;20(22):5621. doi: 10.3390/ijms20225621 (PMC6888598; doi:10.3390/ijms20225621)
Supplement: Supplementary file 1 [file ijms-20-05621-s001.zip › SI_ms_BrCaRnaEdit_17Oct.pdf]

SUPPLEMENTAL MATERIALS

Asaoka et al. *APOBEC3-mediated RNA editing in breast cancer tumors is associated with heightened immune activity and improved survival.*

Content

1. Figure S1. Estimation of RNA editing levels using RNA and DNA sequencing data.
2. Figure S2. Example views of mapped sequencing read alignment data illustrating C-to-U RNA editing events.
3. Figure S3. Correlation of RNA editing levels determined with originally aligned and re-aligned RNA sequencing data.
4. Figure S4. *APOBEC3* gene expression differences between tumors with editing absent and present for four editing sites.
5. Table S1. Genome positions that were examined for RNA editing.
6. Table S2. Characteristics of determinations made for estimation of RNA editing levels.
7. Table S3. Fifty most commonly edited sites among tumors.
8. Table S4. Genes with significant differential expression between editing-high and -low tumors.

Figure S1. *Estimation of RNA editing levels using RNA and DNA sequencing data*. The flow diagram shows the scheme to estimate C-to-U RNA editing level at a given genome position using aligned RNA and DNA sequencing (*seq.*) data. In this diagram, the genome position is in a gene that is transcribed from the + or upper chromosome strand. G and A are respectively the reference and variant bases in case of genes transcribed from the other strand.

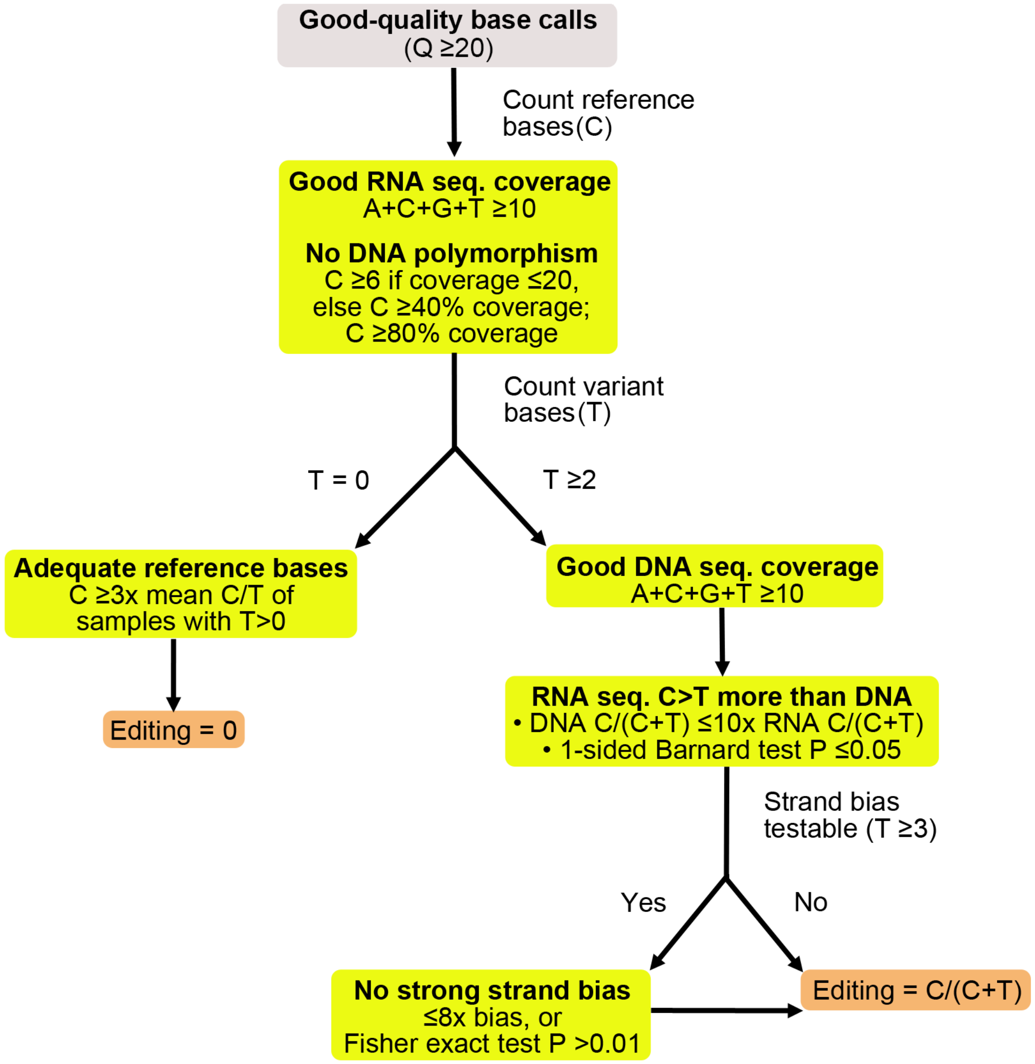

Figure S2. *Example views of mapped sequencing read alignment data illustrating C-to-U RNA editing events*. Screenshots of views in Integrative Genomics Viewer application (version 2.6.3) are shown for two tumor samples each for two C-to-U RNA editing sites (*arrows*) that are in *CYP27A1* and *SERPINA1* genes. These genes are respectively transcribed from the + (upper) and – (lower) chromosome strands. The + strand DNA and translated protein sequences are shown at bottom. Also shown are TCGA patient identifiers, GRCh38 reference-based genome positions, and base calls from aligned RNA or DNA sequencing (*seq.*) reads at the positions. Sequencing reads are in grey with any base mismatch with + strand DNA colored (green: A, red: C). Not all reads are visible in the application window. Read alignments are unfiltered.

Chr. 14:94378482
SERPINA1

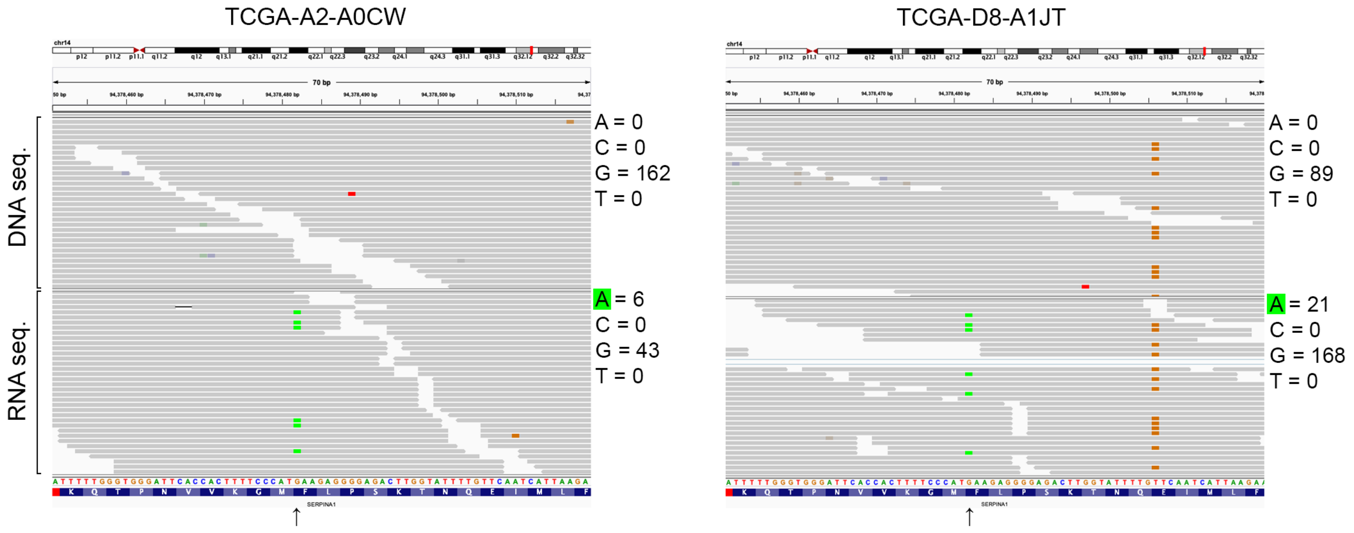

Chr. 2:218812625
CYP27A1

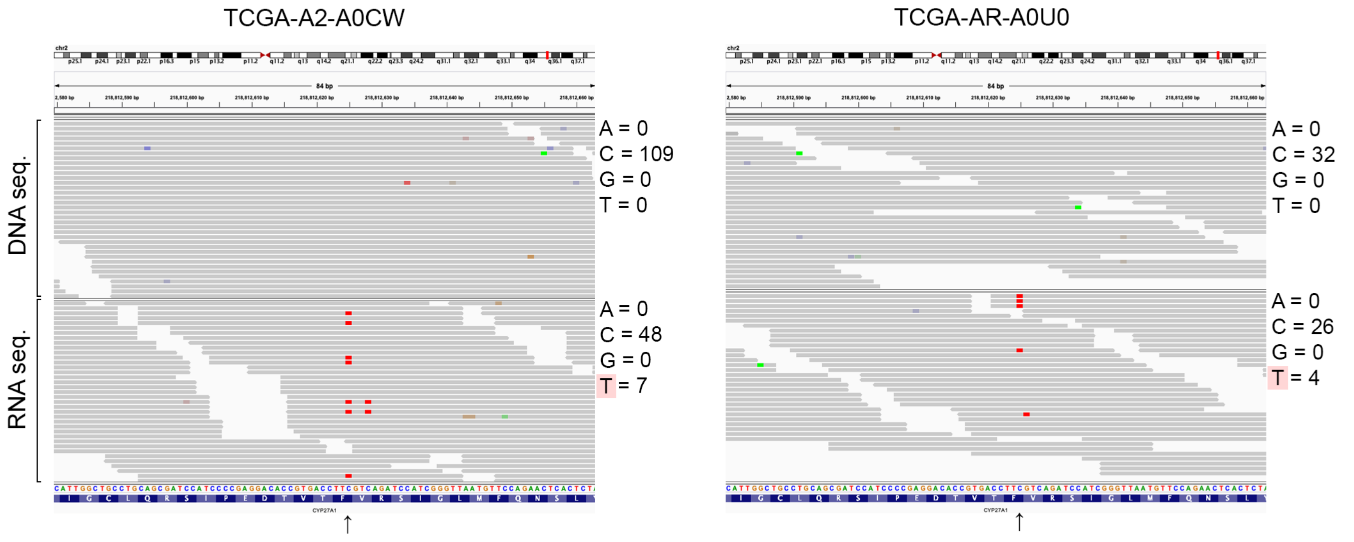

Figure S3. *Correlation of RNA editing levels determined with originally aligned and re-aligned RNA sequencing data.* For 24 C-to-U RNA editing events that were identified in four tumor samples, the RNA editing levels were re-determined after re-aligning RNA sequencing data. For re-alignment, fastq read data was extracted from the aligned data (BAM files) that had been generated by Genomic Data Commons (GDC), and then extracted paired read (but not singletons) were re-aligned using Subread subjunc aligner software against version 81 of the Ensembl GRCh38 reference genome. C-to-U RNA editing levels were calculated as the ratio of variant to sum of variant and reference base calls of phred33 quality ≥ 20. Editing levels with the GDC- and re-aligned data are plotted.

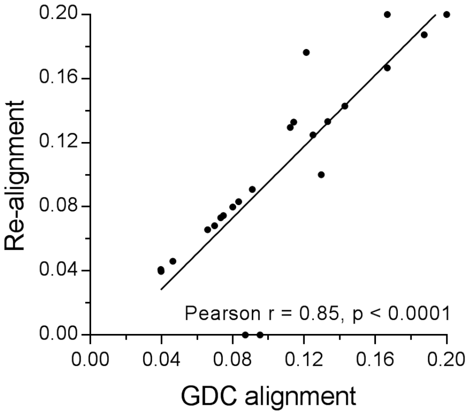

Figure S4. *APOBEC3 gene expression differences between tumors with editing absent and present for four editing sites.* Tukey boxplots show expression of *APOBEC3A*, *APOBEC3G*, and *APOBEC3H* genes in tumors with and without C-to-U RNA editing at four known APOBEC3-mediated RNA editing sites. GRCh38 genome coordinates of the sites and the genes they are in are noted. Shown p values were calculated with standard t test. *TPM*, transcripts per million.

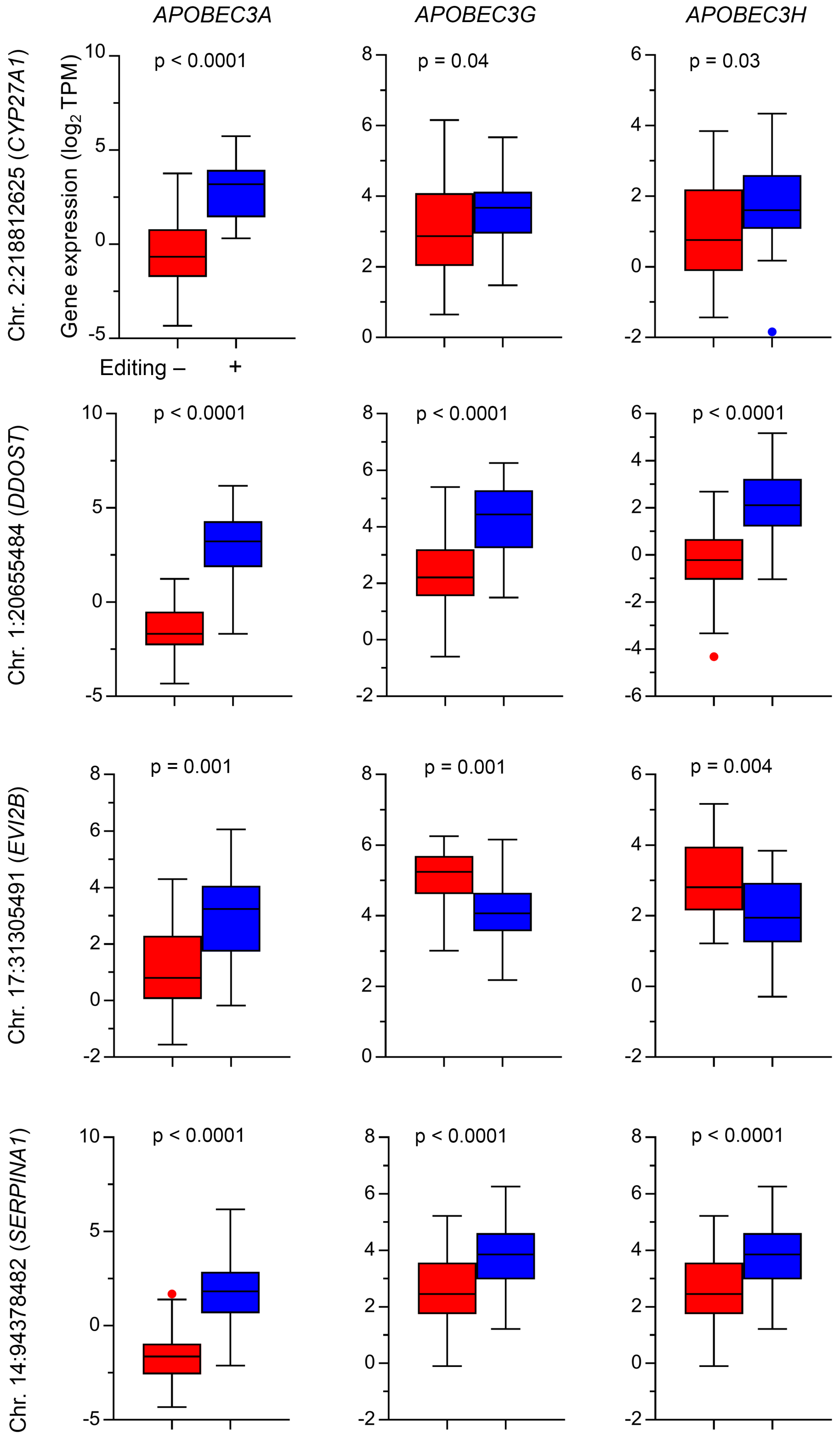

Table S1. Genome positions that were examined for RNA editing[a].

Microsoft Excel file 'Table S1.xls'

[a]Genome coordinates (GRCh38 reference) of the 5,208 C-to-U RNA editing and 198 negative control sites that were examined in the study. Positions for the editing sites are annotated with information such as gene and effect of editing on protein sequence.

Table S2. Characteristics of determinations made for estimation of RNA editing levels[a].

Criterion/feature	*Editing*	*n*	*%*
Poor RNA seq. coverage (< 10):	ND	700,501	11.872
Coverage = 0	ND	154,404	2.617
Coverage = 1-9	ND	546,097	9.255
Likely gene polymorphism[b]:	ND	4,407	0.075
Heterozygous	ND	3,068	0.052
Homozygous	ND	1,339	0.023
0 variant base in RNA seq. data:	ND / 0	4854,111	82.264
Inadequate[b] reference bases	ND	4825,142	81.773
Adequate reference bases	0	28,969	0.491
1 variant base in RNA seq. data	ND	249,075	4.221
> 1 variant base in RNA seq. data:	ND / > 0	92,570	1.569
Poor DNA seq. coverage (< 10):	ND	25,866	0.438
Coverage = 0	ND	8,222	0.139
Coverage = 1-9	ND	17,644	0.299
Good DNA seq. coverage (≥ 10):	ND / > 0	66,704	1.13
P[c] > 0.05 in RNA seq. vs. DNA seq. variation comparison	ND	65,015	1.102
P ≤ 0.05 in RNA seq. vs. DNA seq. variation comparison:	> 0	1,689	0.029
0 variant base in DNA seq. data:	ND / > 0	1,638	0.028
Fail strand-bias test[b]	ND	46	0.001
≥ 1 variant base in DNA seq. data:	ND / > 0	51	0.001
RNA seq. variation leveld < 10× DNA seq.	ND	17	< 0.001
RNA seq. variation level ≥ 10× DNA seq.:	ND / > 0	34	0.001
Fail strand-bias test	ND	1	< 0.001

[a]Determinations were made for 1,133 samples to estimate C-to-U RNA editing levels for 5,208 sites by examination of RNA and DNA sequencing (*seq.*) data using a set of criteria that were applied sequentially, as indicated in this table. The number (*n*) of determinations fulfilling the criteria and their features are listed. Percentages are with respect to the total number of determinations (1,133 × 5,208). Assigned editing level for the criteria and features are also shown (*ND*, not determinable).

[b]Refer to main text for explanation.

[c]In one-sided Barnard test with contingency table of reference and variant base counts in RNA and DNA seq. data.

[d]Variation level is the ratio of variant base count to sum of variant and reference base counts.

Table S3. Fifty most commonly edited sites among tumors.

Genome position	Gene	Tumors			Mean editing level[b] (%)	RNA region[c]	Editing effect
		Editing determinable	Editing present	Edited fraction			
1:153818890	*GATAD2B*	7	7	1.00	4.62	Exonic	Synonymous
14:94378482	*SERPINA1*	198	160	0.81	7.67	Exonic	Synonymous
11:10461666	*AMPD3*	15	12	0.80	12.50	Exonic	Synonymous
17:31305491	*EVI2B*	57	43	0.75	4.59	Exonic	Non-synonymous
10:98418201	*HPS1*	8	5	0.63	8.68	Exonic	Synonymous
12:121162497	*P2RX7*	8	5	0.63	14.67	Exonic	Synonymous
15:22873718	*CYFIP1*	8	5	0.63	8.27	Exonic	Synonymous
16:31381814	*ITGAX*	16	10	0.63	7.39	Exonic	Synonymous
16:12052031	*SNX29*	13	8	0.62	11.68	Exonic	Synonymous
4:109684468	*CCDC109B*	18	11	0.61	3.65	Exonic	Non-synonymous
3:152432796	*MBNL1*	5	3	0.60	5.80	Exonic	Non-synonymous
3:196728061	*PIGX*	17	10	0.59	5.07	Exonic	Non-synonymous
1:155612311	*MSTO1*	12	7	0.58	6.17	Exonic	Non-synonymous
1:161543002	*FCGR3A*	24	14	0.58	2.98	3'UTR	
16:2061900	*TSC2*	7	4	0.57	8.60	Exonic	Synonymous
2:218584705	*RQCD1*	9	5	0.56	3.72	Exonic	Synonymous
3:30672308	*TGFBR2*	9	5	0.56	4.05	Exonic	Synonymous
1:20655484	*DDOST*	109	60	0.55	4.42	Exonic	Synonymous
2:97792880	*TMEM131*	11	6	0.55	4.64	Exonic	Non-synonymous
5:116052036	*ARL14EPL*	61	33	0.54	3.18	Intronic	
1:117096230	*TTF2*	36	19	0.53	9.74	Exonic	Synonymous
1:30873312	*SDC3*	8	4	0.50	6.69	Exonic	Non-synonymous
10:100236856	*CWF19L1*	6	3	0.50	3.22	Exonic	Synonymous
10:109907707	*XPNPEP1*	6	3	0.50	7.22	Exonic	Non-synonymous
11:3691446	*NUP98*	6	3	0.50	3.07	Exonic	Non-synonymous
15:59188146	*MYO1E*	6	3	0.50	4.23	Exonic	Non-synonymous
13:41470511	*RGCC*	62	30	0.48	3.95	3'UTR	
16:47155742	*ITFG1*	23	11	0.48	2.48	Exonic	Non-synonymous
8:123226597	*C8orf76*	13	6	0.46	2.47	Exonic	Non-synonymous
22:29508462	*THOC5*	35	16	0.46	7.35	Exonic	Non-synonymous
9:379811	*DOCK8*	16	7	0.44	12.67	Exonic	Synonymous
10:5927502	*FBXO18*	7	3	0.43	2.37	Exonic	Synonymous
3:53882012	*ACTR8*	7	3	0.43	12.67	Exonic	Synonymous
7:44969774	*MYO1G*	7	3	0.43	8.93	Exonic	Synonymous
15:55360453	*CCPG1*	12	5	0.42	2.65	Exonic	Synonymous
8:89943303	*NBN*	36	15	0.42	2.10	Exonic	Non-synonymous
2:218812625	*CYP27A1*	61	25	0.41	6.14	Exonic	Synonymous
1:1756558	*NADK*	5	2	0.40	3.00	Exonic	Synonymous
1:222664054	*MIA3*	5	2	0.40	2.27	Exonic	Synonymous
11:65183499	*CAPN1*	5	2	0.40	8.10	Exonic	Synonymous
12:123727864	*ATP6V0A2*	5	2	0.40	11.27	Exonic	Synonymous
15:73283052	*NEO1*	5	2	0.40	8.25	Exonic	Synonymous
19:40447937	*BLVRB*	35	14	0.40	1.96	Exonic	Synonymous

2:44322759	*PREPL*	5	2	0.40	1.99	Exonic	Synonymous
20:47310132	*ZMYND8*	5	2	0.40	3.55	Exonic	Non-synonymous
3:23977363	*NR1D2*	5	2	0.40	7.96	Exonic	Stop gain
4:13599665	*BOD1L1*	5	2	0.40	3.30	Exonic	Non-synonymous
5:146271606	*RBM27*	5	2	0.40	8.01	Exonic	Non-synonymous
6:70857991	*SMAP1*	5	2	0.40	7.31	Exonic	Non-synonymous
3:52843751	*TMEM110*	13	5	0.38	4.68	Exonic	Synonymous

[a]Genome coordinates (chromosome:position, GRCh38 reference) of positions for which C-to-U RNA editing was most frequent. Positions with editing level determinable for < 5 tumors are excluded.

[b]Among tumors with editing present.

[c]Annotations for RNA region and effect of editing on protein-coding are generated with ANNOVAR. *UTR*, untranslated region.

Table S4. Genes with significant differential expression between editing-high and -low tumors[a].

Gene[b]	*Name*	*Log2 FC*	*P*	*Adjusted P*
APOBEC3A	apolipoprotein B mRNA editing enzyme catalytic subunit 3A	1.56	7.71E-26	1.68E-21
IDO1	indoleamine 2,3-dioxygenase 1	1.46	1.02E-20	1.31E-17
CXCL11	C-X-C motif chemokine ligand 11	1.43	2.54E-23	7.93E-20
KIR2DL4	killer cell immunoglobulin like receptor, two Ig domains and long cytoplasmic tail 4	1.43	3.14E-18	2.28E-15
CXCL10	C-X-C motif chemokine ligand 10	1.38	1.98E-24	1.44E-20
CASP14	caspase 14	1.37	2.21E-05	2.55E-04
COL6A5	collagen type VI alpha 5 chain	1.37	2.50E-15	6.57E-13
S100A7	S100 calcium binding protein A7	1.36	8.48E-05	7.55E-04
IFNL1	interferon lambda 1	1.32	1.88E-15	5.25E-13
CXCL9	C-X-C motif chemokine ligand 9	1.30	1.34E-15	4.10E-13
ZBED2	zinc finger BED-type containing 2	1.28	1.91E-15	5.28E-13
S100A8	S100 calcium binding protein A8	1.28	5.69E-10	3.77E-08
IFNL2	interferon lambda 2	1.27	2.67E-15	6.93E-13
GZMB	granzyme B	1.26	9.44E-18	5.72E-15
AQP9	aquaporin 9	1.26	2.02E-14	4.20E-12
CXCL13	C-X-C motif chemokine ligand 13	1.24	1.93E-09	1.09E-07
LAMP3	lysosomal associated membrane protein 3	1.23	1.90E-20	2.18E-17
IFNG	interferon gamma	1.23	1.60E-12	2.19E-10
LINC02446	long intergenic non-protein coding RNA 2446	1.23	7.96E-13	1.17E-10
ICOS	inducible T cell costimulator	1.20	5.49E-17	2.44E-14
CLEC6A	C-type lectin domain containing 6A	1.20	7.24E-13	1.07E-10
GBP1P1	guanylate binding protein 1 pseudogene 1	1.19	7.29E-22	1.32E-18
GBP5	guanylate binding protein 5	1.19	3.59E-19	3.27E-16
S100A7A	S100 calcium binding protein A7A	1.18	4.71E-05	4.68E-04
ACOD1	aconitate decarboxylase 1	1.17	2.89E-14	5.63E-12
ADAMDEC1	ADAM like decysin 1	1.17	1.29E-14	2.81E-12
MARCO	macrophage receptor with collagenous structure	1.16	1.28E-09	7.55E-08
PRAME	preferentially expressed antigen in melanoma	1.16	3.24E-04	2.22E-03
IGHV2-70	immunoglobulin heavy variable 2-70	1.15	4.45E-07	1.01E-05
IFNB1	interferon beta 1	1.14	1.68E-15	4.82E-13
A2ML1	alpha-2-macroglobulin like 1	1.13	2.37E-07	6.01E-06
KLHDC7B	kelch domain containing 7B	1.11	1.03E-13	1.78E-11
CCR8	C-C motif chemokine receptor 8	1.10	4.01E-12	4.95E-10
FPR2	formyl peptide receptor 2	1.10	5.11E-15	1.22E-12
TNIP3	TNFAIP3 interacting protein 3	1.10	3.62E-14	6.93E-12
GBP6	guanylate binding protein family member 6	1.10	2.61E-17	1.27E-14
AIM2	absent in melanoma 2	1.09	5.43E-18	3.70E-15
UBD	ubiquitin D	1.09	1.80E-10	1.37E-08
OASL	2'-5'-oligoadenylate synthetase like	1.09	4.57E-22	9.07E-19
CD38	CD38 molecule	1.09	2.36E-15	6.33E-13
CCL18	C-C motif chemokine ligand 18	1.08	4.73E-09	2.27E-07
IFI44L	interferon induced protein 44 like	1.08	7.40E-21	1.08E-17
IL22RA2	interleukin 22 receptor subunit alpha 2	1.08	8.22E-08	2.51E-06
TIGIT	T cell immunoreceptor with Ig and ITIM domains	1.08	1.22E-15	3.90E-13
MAGEA4	MAGE family member A4	1.07	3.25E-05	3.48E-04

XIRP1	xin actin binding repeat containing 1	1.07	1.95E-11	1.99E-09
MMP1	matrix metallopeptidase 1	1.07	1.09E-07	3.22E-06
CCL8	C-C motif chemokine ligand 8	1.06	2.46E-17	1.22E-14
CARD17	caspase recruitment domain family member 17	1.06	1.64E-10	1.27E-08
NCCRP1	NCCRP1, F-box associated domain containing	1.06	7.73E-07	1.58E-05
IFNL3	interferon lambda 3	1.05	7.88E-11	6.82E-09
RSAD2	radical S-adenosyl methionine domain containing 2	1.05	4.18E-23	1.14E-19
PLA2G2D	phospholipase A2 group IID	1.04	6.70E-08	2.10E-06
IFI27	interferon alpha inducible protein 27	1.03	7.61E-16	2.54E-13
P2RY10	P2Y receptor family member 10	1.03	2.00E-14	4.20E-12
IGHV2-5	immunoglobulin heavy variable 2-5	1.03	2.85E-06	4.79E-05
CTLA4	cytotoxic T-lymphocyte associated protein 4	1.02	2.43E-14	4.86E-12
OAS2	2'-5'-oligoadenylate synthetase 2	1.02	1.61E-24	1.44E-20
IGF2BP3	insulin like growth factor 2 mRNA binding protein 3	1.01	3.18E-09	1.63E-07
CCL13	C-C motif chemokine ligand 13	1.01	1.02E-09	6.22E-08
CSAG3	CSAG family member 3	1.01	1.37E-06	2.56E-05
LINC01281	long intergenic non-protein coding RNA 1281	1.01	1.12E-09	6.76E-08
ZBP1	Z-DNA binding protein 1	1.01	2.90E-16	1.07E-13
ACTL8	actin like 8	1.01	1.32E-04	1.08E-03
IL21	interleukin 21	1.00	3.69E-12	4.60E-10
MUC15	mucin 15, cell surface associated	1.00	1.31E-04	1.07E-03
TFF1	trefoil factor 1	-1.42	4.77E-05	4.74E-04
KCNJ3	potassium inwardly rectifying channel subfamily J member 3	-1.28	1.85E-04	1.42E-03
TFF3	trefoil factor 3	-1.21	6.42E-06	9.23E-05
CST9	cystatin 9	-1.20	3.78E-04	2.51E-03
SERPINA11	serpin family A member 11	-1.18	1.51E-05	1.86E-04
SERPINA6	serpin family A member 6	-1.18	5.05E-04	3.15E-03
PHF21B	PHD finger protein 21B	-1.18	9.81E-09	4.19E-07
TPSG1	tryptase gamma 1	-1.15	9.32E-07	1.85E-05
CYP2B7P	cytochrome P450 family 2 subfamily B member 7, pseudogene	-1.14	2.36E-04	1.71E-03
MAPT-AS1	MAPT antisense RNA 1	-1.12	6.07E-06	8.83E-05
SRARP	steroid receptor associated and regulated protein	-1.09	3.91E-04	2.57E-03
AGR3	anterior gradient 3, protein disulphide isomerase family member	-1.08	2.77E-04	1.95E-03
MAPT-IT1	MAPT intronic transcript 1	-1.08	5.10E-06	7.70E-05
ABCC8	ATP binding cassette subfamily C member 8	-1.07	8.30E-06	1.14E-04
WNK4	WNK lysine deficient protein kinase 4	-1.07	7.08E-08	2.21E-06
LINC01411	long intergenic non-protein coding RNA 1411	-1.06	2.76E-06	4.67E-05
LINC02224	long intergenic non-protein coding RNA 2224	-1.05	4.01E-04	2.62E-03
GRIA2	glutamate ionotropic receptor AMPA type subunit 2	-1.04	4.05E-04	2.64E-03
PLPPR3	phospholipid phosphatase related 3	-1.02	3.87E-06	6.19E-05
NXPH1	neurexophilin 1	-1.01	8.73E-04	4.86E-03

[a]Genes are ordered by fold-change (*FC*) value (editing-high vs. -low). P values in limma's t test were adjusted with Benjamini-Hochberg method. Genes with ≥ 2 fold-change and adjusted P < 0.05 are listed.

[b]Human Genome Organization symbols and names are used.
